# The Output of Defectives

**Published:** 1912-04

**Authors:** 


### EDITORIAL DEPARTMENT

DR. F. E. DANIEL, Editor DR. R. H. L. BIBB, Associate Editor

### THE OUTPUT OF DEFECTIVES.

The world seems to be awakening to a realization of the fact that under our mistaken notions of humanity,—"humanity to the individual,—inhumanity to the race," there is an over-production of undesirables, and steps to arrest the output, to some extent at least, are being advocated by medical men and taken by several States; and "sterilization" is the slogan note. For instance: in Texas the insane are increasing out of all proportion to population, and faster than the State can make provision for their care. This increase is largely due to hereditary transmission of insanity. Our asylums are always so crowded that the chronics—males—are furloughed, and allowed to go home. In a few years their progeny succeeds them in the asylum. The cost to the State for the care of the insane was one million dollars in 1910. Seven States have passed laws for the purpose of cutting off this excessive production of insane and criminals, and I publish herewith that of Oregon, the latest and best, kindly furnished by that zealous and able reformer, Dr. Bogart, with whose strong papers the readers of the "Red Back" are familiar. The movement is growing. Vasectomy—by whom discovered and first practiced I do not know—was an inspiration. It "solved the problem." Castration is now out of date, though prior to vasectomy it was urged and recommended to lawmakers as the only method then known whereby procreation could be prevented. But it was too radical. While it prevented impregnation, it also unsexed and mutilated the subject. It was dangerous, and it was claimed that it was unlawful,—constituting the crime of mayhem, and unconstitutional in that it inflicted "cruel and unusual punishment"—so claimed by those who have the making of the laws. By vasectomy these objections are obviated.

Castration of Sexual Perverts.—Nearly twenty years ago, towit, in August, 1893, the editor of this Journal presented a paper under the above title at a joint meeting of the Medico-Legal Society of New York and that of Chicago, during the World's Congress meeting in Chicago, at the quadri-centennial of the discovery of America, and its publication in the secular press created a sensation. In most quarters it was laughed at, but the seed then sown has borne fruit, and today seven States have adopted and put in operation the essential means—to the identical ends then proposed—sterilization of the male degenerates, including insane and criminal. This paper was extensively published in the medical press and in the Medico-Legal Journal; but it was so long ago, and is now so little known, that feeling that I have some claim to priority (so far as I know), I am tempted to reproduce it in this issue of the Journal. It appears under head of "Original Articles."

